# Modulation of the Product Upon the Reaction of CO_2_ With Dimethylamine Cluster: A Topological Analysis of the Reaction Mechanism

**DOI:** 10.1002/jcc.70135

**Published:** 2025-05-14

**Authors:** Mohammad Esmaïl Alikhani, Bernard Silvi

**Affiliations:** ^1^ MONARIS, CNRS‐UMR 8233 Sorbonne Université Paris France; ^2^ LCT, CNRS‐UMR 7616 Sorbonne Université Paris France

**Keywords:** carbamate, carbamic acid, CO_2_ capture, non‐reductive activation, tetrel bond

## Abstract

The capture, activation, and reaction of carbon dioxide with dimethylamine (DMA) clusters have been investigated theoretically in the gas phase. The electronic structure of various compounds has been obtained using the density functional theory approach. The partitioning of the reaction path into different domains of structural stability has been done within the framework of the electron localization function (ELF) analysis. It has been found that DMA cluster size is a key parameter in modulating CO_2_ conversion, both energetically and structurally. It has been shown that as the size of DMA clusters increases, hidden domains transform into real domains and the energy barrier for the rate‐limiting step significantly decreases, so that a slow and unlikely reaction becomes an instantaneous and viable reaction. Carbamic acid hydrogen bonded with a DMA dimer is the unique product of the reaction of CO_2_ with a DMA trimer.

## Graphical abstract

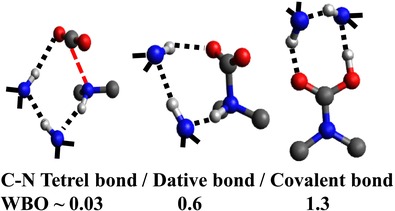

## 1 Introduction

Although carbon dioxide is a vital compound for plant life on earth, the rapid increase of atmospheric CO_2_ concentrations has undesired consequences for climate change. Global warming, atmospheric CO_2_ levels, and world population are interconnected. The capture of carbon dioxide and its transformation into useful and valuable chemicals, advanced materials, and energy are long‐standing challenges in fundamental science and industry [1, 2, 3, 4, 5, 6, 7, 8, 9, 10]. The activation of CO_2_ molecules, a key step in chemical CO_2_ conversion, could be achieved via either a reduction/reductive functionalization or a non‐reduction reaction of CO_2_ [11, 12, 13]. Although CO_2_ reduction is mainly accomplished using transition metal‐based catalysts [12, 14, 15, 16, 17, 18], metal‐free alternatives have also been suggested [11, 13, 19, 20]. For the non‐reductive CO_2_ transformation, with no formal C (or O) redox, the CO_2_ activation mainly occurs in the Lewis acid–Lewis base pairs, where a specific acidity/basicity strength is likely required [1, 9, 10, 17, 20, 21, 22, 23, 24, 25, 26, 27, 28]. CO_2_ activation is typically indicated by a significant geometric distortion—an ∠OCO angle of ~135° —reflecting in lowering the energy of its lowest‐energy unoccupied molecular orbitals (LUMOs), especially for the in‐plane one [11, 12, 13, 29]. These changes enhance the electron‐accepting ability of CO_2_. Consequently, the one‐electron reduction of CO_2_ may best be formulated as CO_2_^− •^, while non‐reductive activated CO_2_ results in the formation of a dative bond (acceptor–donor bond) between an electron pair donor (such as nucleophilic amine group) and an electrophilic carbon center Nuc:→CO_2_.

To transform atmospheric CO_2_ into biomass, Nature uses an enzyme (Rubisco) [30] able to fix and activate atmospheric CO_2_ during photosynthesis reactions thanks to an amine group (nucleophile) present in the Rubisco active site. Inspired by nature, numerous research studies have been devoted to CO_2_ conversion mainly using amines [25, 28, 31, 32, 33, 34, 35, 36, 37]. It has been shown that the interaction between a primary or secondary amine and the CO_2_ molecule, due to the synergistic action of the electron donation (from the amine nitrogen lone pair to the electrophilic CO_2_ carbon atom) and the hydrogen bond (from the aminic proton to the CO_2_ oxygen atom), can lead, with more or less acceptable activation energy, to the formation of a carbamic acid or carbamate anion [1, 10, 25, 26, 27, 28, 31, 32, 34, 35, 36, 37, 38, 39].

Since dimethylamine (DMA) is in the gas phase at room temperature, there is no experimental study on the interaction of CO_2_ with DMA. Only the CO_2_ absorption in amine solutions has been studied experimentally: either in non‐aqueous amine sorbents such as dipropylamine (DPA) [10, 22, 23, 27] or in aqueous solution. For the latter, we can cite the case of methylamine‐carbon dioxide complexes (CH_3_NH_2_/CO_2_) relevant for the chemical transformation of ice grains in the interstellar medium [40].

In this paper, we have undertaken the interaction between CO_2_ and DMA cluster as a model system, a non‐computationally prohibitive model, for academic purposes to unravel the characteristic features of the reaction mechanisms.

Due to its basicity and acidity, dimethylamine molecules easily form hydrogen‐bonded DMA clusters [41]. The first two clusters, DMA dimer and trimer, have, respectively, a chain geometry (*C*_*s*_ symmetry) and a cyclic structure [42, 43].

This work focuses on the study of the following three reactions:

$$\text{R1}:{\left({\mathrm{CH}}_{3}\right)}_{2}\mathrm{NH}+{\mathrm{CO}}_{2}={\left({\mathrm{CH}}_{3}\right)}_{2}\text{NCOOH}.$$

$$\text{R2}:{\left[{\left({\mathrm{CH}}_{3}\right)}_{2}\mathrm{NH}\right]}_{2}+{\mathrm{CO}}_{2}={\left({\mathrm{CH}}_{3}\right)}_{2}\text{NCOOH}+{\left({\mathrm{CH}}_{3}\right)}_{2}\mathrm{NH}.$$

$$\text{R3}:{\left[{\left({\mathrm{CH}}_{3}\right)}_{2}\mathrm{NH}\right]}_{3}+{\mathrm{CO}}_{2}={\left({\mathrm{CH}}_{3}\right)}_{2}\text{NCOOH}+{\left[{\left({\mathrm{CH}}_{3}\right)}_{2}\mathrm{NH}\right]}_{2}.$$

Chemical compounds with the formula (CH_3_)_2_NH, [(CH_3_)_2_NH]_2_, [(CH_3_)_2_NH]_3_, and (CH_3_)_2_NCOOH correspond to DMA, DMA dimer, DMA trimer, and carbamic acid, respectively.

The main questions to be addressed in the current investigation are as follows:

1. For the three reactions mentioned above, what are the stationary points (minima and transition stats) along the reaction pathway?
2. Are the three chemical reactions energetically viable? Can we deduce the minimum number of DMA required to convert CO_2_ into carbamic acid?
3. What are the most relevant chemical events (bond‐breaking/forming processes) that characterize the reaction dynamics along the reaction pathway?

## 2 Computational Details

All first‐principles calculations were performed using the Gaussian 09 quantum chemical packages [44]. Optimization of the stationary points on the potential energy surface was performed using the ωB97XD hybrid range‐separated exchange‐correlation functional [45, 46] which accounts for dispersion energy and long‐range interaction. The Pople triple‐ζ quality basis set extended with polarization and diffuse functions, 6–311++G(2d, 2p) has been used for all atoms [47, 48].

Although the reliability of this functional is already highlighted in the literature [49], we performed some additional calculations to show the accuracy and reliability of this functional when studying the system in hand: optimization of all the structures of the reaction R1 at the second Møller–Plesset perturbation MP2 level of theory, as included in the Gaussian 09 codes [44].

At this point, we would like to present our strategy to choose the starting structure of a reaction mechanism. For three reactions studied in this work, any mechanism leading to the formation of a C‐N bond followed by a proton transfer necessarily implies the upstream formation of a dative C‐N bond. Having identified the structure supporting a dative C‐N bond, we searched to optimize the structure of the CO_2_:(DMA)_1–3_ complex, which would be linked to the dative structure by a transition structure. Indeed, this complex was chosen as the first structure to build the reaction pathway. We note that this first structure corresponds to the complex formed between CO_2_ and DMA in the case of the reaction R1, and to the complex formed between CO_2_ and the DMA dimer in the case of the reaction R2. For the reaction R3, on the other hand, the starting structure is a cyclic complex with four molecules (one carbon dioxide and three dimethylamine molecules) linked in pairs by non‐covalent interactions, like the DMA tetramer. After finding a dative structure (Dat3 in the reaction R3, for example), we sought to determine the structure of the product, i.e., the carbamic acid in interaction by hydrogen bonding with amines (Trans‐P3 in the reaction R3, for example). Other structures of the reaction path are simply constructed by the successive proton transfer between the dative structure and that of the product. (Vide infra).

Calculation of the reaction path was performed by following the intrinsic reaction coordinate (IRC) [50] labeled as ξ (amu^1/2^·Bohr) and expressed in mass‐weighted Cartesian coordinates, which links the transition state TS (characterized by a single imaginary frequency) to the reactant and product. All IRC calculations have been performed using the local quadratic approximation (LQA) algorithm [51, 52].

The rate constant, k(T), was calculated using the Eyring–Polanyi equation, k(T) = (k_B_T/*h*)·exp.(−ΔG^‡^/RT) where k_B_, *h*, and R are Boltzmann's constant, Planck's constant, and the universal gas constant, respectively. ΔG^‡^ is the standard‐state free energy of activation, ΔG^‡^ = ΔG°(TS) ‐ ΔG°(reactant).

We used a natural bond orbital descriptor—namely the Wiberg bond index—to describe the chemical bonding nature going from the C···N tetrel bond to the C–N dative bond [53].

The chemical reaction force, along the reaction path, was calculated as the opposite of the first derivative of the potential energy with respect to the intrinsic reaction coordinate, F = − dE(ξ)/dξ [54, 55].

To analyze the evolution of chemical bonds along the reaction path, we used the electron localization function (ELF) [56, 57]. Indeed, the ELF topology provides a partitioning of the molecular space into chemically representative regions (basins of attractors) corresponding to the chemical object in the framework of the Lewis valence theory and also in the valence shell electron pair repulsion (VSEPR) approach [58].

In this work, the partition of the molecular space in terms of non‐overlapping space‐filling domains has been performed using the TopMod package [59].

The bond evolution theory (BET) [60, 61, 62, 63], based on the ELF topology of reorganization of covalent bonds and lone pairs along the reaction path, allowed us to identify and describe most of the electronic events (such as bond breaking/forming and electronic density redistribution) that occur along the reaction path.

## 3 Results and Discussion

The energetics, selected reaction properties, and the most relevant topological events describing the chemical bond evolution along the reaction path are presented for each reaction R1–R3. The Cartesian coordinates of all stationary points can be found in the Supporting Information.

Let us clarify one point before beginning the discussion of the full results.

In order to check the reliability of the wB97XD functional in the study of CO_2_ reduction by dimethylamine cluster, we also optimized the five structures of the reaction R1 at the MP2/6‐311 level. The most relevant geometric and energetic results are presented in Figures SI‐1 and SI‐2 (see Supporting Information). The calculated mean absolute errors (MAE) on the bond lengths (MAE(r) = 0.009 Å) and on the reaction enthalpies (MAE(ΔH^°^) = 0.7 kcal/mol) confirm the reliability of the wB97XD functional used for this work.

### 3.1 Energetics

Over the last two decades, several studies have shown that the activation and conversion of CO_2_ by a primary amine is a very slow and unlikely reaction because of a very high activation barrier [23, 27, 28, 64, 65, 66, 67]. In agreement with previous studies, our investigation showed that the R1 reaction is very slow and unlikely because its rate constant is close to zero (k(298) = 1.2 × 10 ^−15^ s^−1^) [39]. In contrast, the R2 reaction has a large rate constant (k(298) = 1.4 × 10^4^ s^−1^) when studying the transformation of the rate‐limiting step, i.e., Dat2 to Cis‐P2 (see Figure SI‐1). Unlike reaction R1, the formation of Cis carbamic acid occurs, with the assistance of a second amine, in two steps within reaction R2.

**FIGURE 1:**
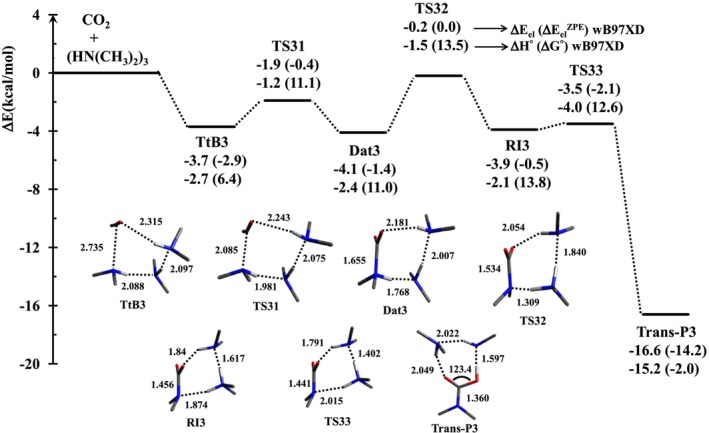
Relative energies (ΔE) of four minima and three transition states found for the reaction R3 are listed below each structure: Relative electronic energy (ΔE_el_) and electronic energy corrected for the zero‐point energy (ΔE_el_^ZPE^) in the first line, and enthalpy (ΔH^°^) and free energy (ΔG^°^) in the second line. Geometries of local minima and transition states are also included.

Figure 1 illustrates the relative energy (ΔE in kcal/mol) of all optimized structures (minima and transition states) on the potential energy surface (PES) for reaction R3.

Reaction R3 occurs through three elementary steps, the first of which is the rate‐limiting step: from TtB3 to Dat3. Note that the third step, corresponding to the transformation of Dat3 into RI3, is not yet sufficiently stabilized; it disappears when we take into account the thermal correction of the total energy of the system (ΔH^°^ instead of ΔE_el_). The assistance of a third amine decreases the activation energy for the rate‐limiting step (6.4 kcal/mol) and thus significantly increases the rate constant (k(298) = 2.2 × 10^9^ s^−1^).

**FIGURE 2:**
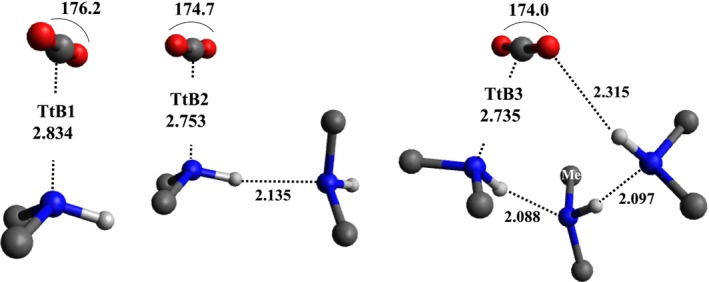
Tetrel bonded TtBn (*n* = 1–3) structures for CO_2_ + *n*(DMA) clusters. Bond lengths are in angstroms and ∠OCO angle in degrees. For clarity, we have omitted the methyl group hydrogens.

The π‐hole driven tetrel bond is a non‐covalent interaction between an electrophilic carbon and a nucleophilic center (nitrogen lone pair) [39, 68, 69, 70, 71, 72], with a moderate interaction energy, ~4 kcal/mol. In tetrel‐bonded structures (Figure 2), the Lewis acid–Lewis base distance and ∠OCO bond angle slightly decrease as the number of DMA increases within the hydrogen‐bonded DMA cluster.

Interestingly, the slightly bent structure of CO_2_, due to the formation of the C···N tetrel bond, is just the beginning of the non‐reductive activation of CO_2_. In this regard, it should be noted that the π‐hole driven interaction is enhanced by the polarization effect exerted by the nucleophilic site [73, 74].

It is worth noting that the calculation has been carried out for isolated species, neglecting therefore the effects of the environment. In most models, such as Onsager's, the energy lowering is roughly proportional to the square of the dipole moment. The CO_2_ + DMA relative energy and dipole moment profiles along the IRC displayed in Figure SI‐3 have very similar behaviors, and therefore a significant lowering of the TS energy should be expected with a polar solvent, such as DMA. Self Consistent Reaction Field calculations have been performed for the tetrel complex, the TS geometry, and the dimethyl carbamic acid, with the DMA dielectric constant ε = 36.70 yielding stabilization energies of 0.48, 4.34, and 5.26 kcal/mol. This trend is in line with previous studies on the proton transfer reaction assisted by solvent molecules [75, 76].

### 3.2 Reaction Mechanism

#### 3.2.1 Reaction R1

We recall that reaction R1 is a two‐step reaction: it first goes from the tetrel‐bonded complex to the cis‐product via an activated complex requiring a barrier height of ~33 kcal/mol, and then from the cis‐product to the trans‐product via an energy barrier of ~4 kcal/mol. The second step occurs simply by rotating the COOH group from the cis‐ to the trans‐position. We have not identified any topological evolution during this step. On the contrary, topological changes occur during the first step of the reaction R1. Therefore, we limit ourselves to describing only the first step of the reaction.

**FIGURE 3:**
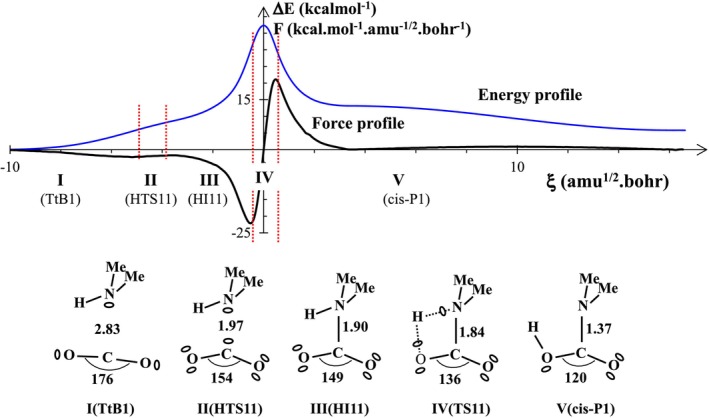
Energy and reaction force profiles, ELF topological regions, and the most relevant ELF bonding representation along the reaction path covering the first step of the reaction R1 connecting the tetrel‐bonded complex (TtB1) to the cis‐Product. Note that “Me” stands for methyl group. Bond lengths are in angstroms and ∠OCO angle in degrees.

Selected reaction properties along the first step obtained from the ELF topological analysis of reaction R1 are presented in Figure 3.

A careful examination of the energy profile reveals the presence of an almost broad (from ξ ≅ −3 to −6 amu^1/2^·Bohr) and flat shoulder on the left side of the reaction path. The same singularity persists on the reaction force profile in Figure 3 (black line). We obtained an unambiguous description of the molecular mechanisms, using the ELF topology to identify all the structural stability domains along the reaction pathway. As illustrated in Figure 3, five domains of structural stability were identified along the reaction path. It should be noted that a topological structure representative of a domain of structural stability should not be confused with a stationary point in the PES. An optimized structure in the PES could, of course, represent a topological structure, provided that its geometric parameters are considered for a given point on the PES. Accordingly, three optimized structures (labeled as TtB1, TS11, and cis_P1 in Figure 3) are representative structures for three corresponding topological domains, namely domains I, IV, and V. The other two domains (labeled as II and III in Figure 3) reveal a hidden transition state structure (labeled as HTS11) which leads to a hidden intermediate region (labeled as HI11). The “hidden intermediate” and “hidden transition state” are two powerful concepts, suggested two decades ago by Cremer and Kraka [77, 78, 79] and also by other researchers [80, 81, 82, 83], to explain some singularities in the case of Diels–Alder reactions, in particular, and for other types of reactions. Indeed, a hidden intermediate does not correspond to any optimized structure. Nevertheless, it could be evidenced as a real species (a minimum in the PES) under other reaction conditions [81, 84]. The topological domain represented by the optimized TS11 structure connects the third domain to that of the cis product (domain V). Although hidden, the 3rd domain is where a covalent bond is formed between the nitrogen of DMA and the carbon of CO_2_. In the transition state domain (IV), well delimited by the extrema of the reaction force (see force profile in Figure 3), a four‐membered mechanism takes place involving a roaming (delocalized) proton [85, 86] located between two electronegative atoms. The roaming proton carries a tiny negative charge (≈ 0.3 e) [87, 88]. It is worth noting that the particularly high transition state simultaneously reflects two structural phenomena: the transition from the C···N tetrel bond to the C‐N shared electron bond and the transfer of the aminic proton to the COO^−^ group. Finally, we should note that the C‐N bond distance and the ∠OCO bond angle continuously decrease along the reaction path: the first goes from 2.83 to 1.37 Å, and the second from 176° to 120°. The C‐N bond length within the cis‐product, 1.37 Å, is characteristic of a simple covalent bond.

**FIGURE 4:**
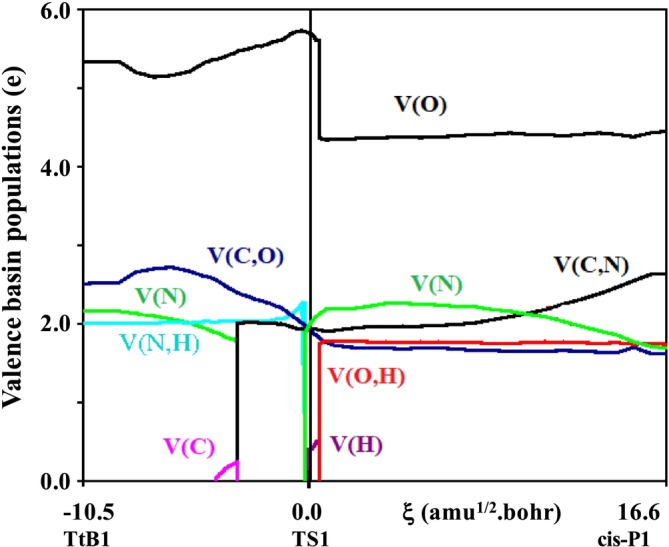
The valence ELF electron density flows along the IRC for the CO_2_ + DMA reaction. Electron lone pairs are represented by V(N), V(O)and V(C) basins, while V(O,H), V(C,N), V(N,H), and V(C,O) stand for the bonding basins. V(H) stands for the tiny negative charge borne by proton.

Electron density flows along the IRC for the reaction R1 represented in Figure 4, indicating that the V(C) population results from the division of V(N) rather than from a transfer from the CO_2_ moiety. Therefore, the first step of the reaction should be interpreted as the formation of a dative bond from a tetrel secondary interaction.

#### 3.2.2 Reaction R2

**FIGURE 5:**
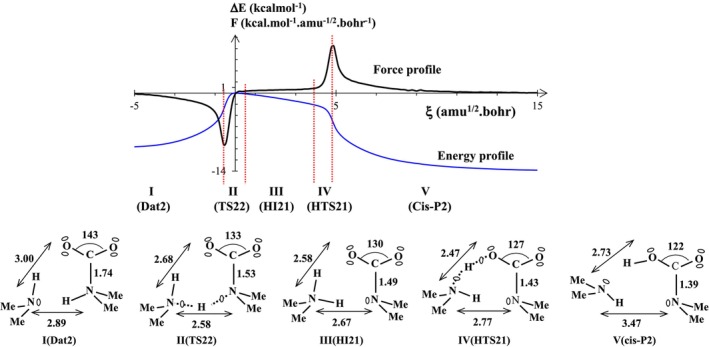
Energy and reaction force profiles, ELF topological regions, and the most relevant ELF bonding representation along the reaction path for the reaction R2 connecting the dative‐bonded complex (Dat2) to the cis‐product. Note that “Me” stands for methyl group. Bond lengths are in angstroms and ∠OCO angle in degrees.

The three‐step reaction R2 gives rise to three transition states (TS21, TS22, and TS23), a reactant (TtB2), a product (Trans‐P2), and two reaction intermediates (Dat2 and Cis‐P2). In the first step, the C···N tetrel bond transforming into a C‐N dative bond shows no topological change during this transformation [89]. However, as has been widely discussed in the past [90, 91], this transformation can be explained in terms of the Wiberg Bond Order (WBO) index described within the NBO code [92]. In this transformation, the WBO index changes from ~0.03 (typical value for the tetrel bond) to ~0.6, characteristic of a dative bond. Furthermore, since the third step (from cis‐P2 to trans‐P2) does not exhibit any structural changes, it is not discussed here. We therefore limit our study to dissecting the second step in reaction R2, where the dative structure transforms into the cis‐product (Figure 5). The energy profile (blue line) exhibits a wide shoulder (~4 amu^1/2^·Bohr) on the product side (plateau‐like) which results from the strong asynchronicity of the reaction in this step. We also observe this singularity on the force profile (black line) whose extrema are connected by a plateau region going from ξ ≅ 0.5 to 4 amu^1/2^·Bohr. According to the suggestion of one of the reviewers, we calculated the energy diagram of the second step of the reaction R2 at the MP2 level of theory (see Figure SI‐4). The activation energy (~14 kcal/mol) and the presence of a pseudo‐plateau on the “forward IRC” side obtained at the MP2 level confirm those found with the wB97XD functional.

Some selected distances as well as the OCO angle are reported on the structures (see Figure 5 bottom). These values represent only a given situation (a snapshot) and more or less vary within a domain, while the topological structure of the organization of lone pairs and bonding doublets does not change.

As illustrated in Figure 5, five different domains of structural stability (I‐V) have been identified along the reaction path. During this step, two proton transfer reactions allow the passage from the reactant domain I (Dat2) to the product region V (cis‐product). The first domain, going from ξ ≅ −5 to −1 amu^1/2^·Bohr is characterized by a topological structure similar to the Dat2 structure, which corresponds to a local minimum. The optimized transition state (TS22) represents the second topologically stable domain, ranging from ξ ≅ −5 to −1 amu^1/2^·Bohr. The third and fourth domains of structural stability correspond to the hidden intermediate region (HI21) and the hidden transition state region (HTS21) which do not match, by definition, any stationary points. Interestingly, the chemical compound in domain III is a zwitterionic compound, in which the carbamate (CH_3_)_2_NCOO^−^ is one of the two constituents (see structure HI21 in Figure 5 bottom).

The fifth domain can be perfectly represented by the optimized structure of cis‐Product. Therefore, it is interesting to highlight the fact that the modification of the DMA cluster size, dimer instead of monomer, allowed us to reveal an additional structure, that of the dative complex, in the entrance channel, and a hidden intermediate region just before the cis‐product. As shown in Figure 5 (bottom), the bond distance characteristic of the evolution of a reactant to the product is smaller at the transition state than for the reactant. For instance, the N‐N distance changes from 2.89 to 2.58 Å when going from Dat2 to TS22.

#### 3.2.3 Reaction R3

As illustrated in Figure 1, the three‐step reaction R3 gives rise to three transition states (TS31, TS32, and TS33), a reactant (TtB3), a product (Trans‐P3), and two reaction intermediates (Dat3 and RI3). In the first step, the C···N tetrel bond transforms into a C‐N dative bond. We skip this step and subsequently focus our ELF topological analysis on the second and third steps along the reaction path.

**FIGURE 6:**
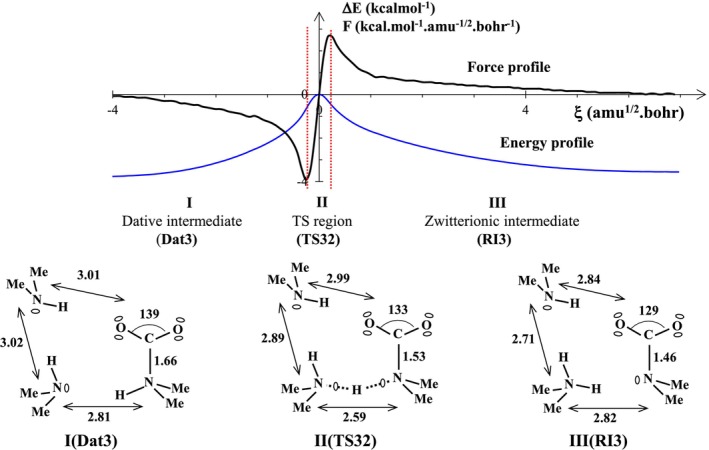
Energy and reaction force profiles, ELF topological regions, and the most relevant ELF bonding representation along the reaction path covering the second step of the reaction R3 connecting the dative‐bonded complex (Dat3) to the first zwitterionic intermediate (RI3). Note that “Me” stands for methyl group. Bond lengths are in angstroms and ∠OCO angle in degrees.

As shown in Figure 6, the transformation of the reactant Dat3 to the product RI3 occurs via a perfectly synchronous process (force profile without shoulder), and with a moderate energy input, ~4 kcal/mol. The reaction pathway is decomposed into three topologically stable domains which are represented by three optimized structures: the dative‐bonded reactant (Dat3), the transition structure (TS32), and the product which is only a reaction intermediate (RI3). In the transition state, the N‐N distance evolving during this transformation decreases by 0.2 Å compared to the value reported for the reactant (2.81 Å). This decrease is the geometric signature of the presence of a roaming proton stabilized by two electronegative centers. The product of this transformation is a zwitterionic compound in which the carbamate (CH_3_)_2_NCOO^−^ species is one of the two constituents, whereas the cationic partner is a protonated DMA dimer, (DMA)_2_H^+^.

**FIGURE 7:**
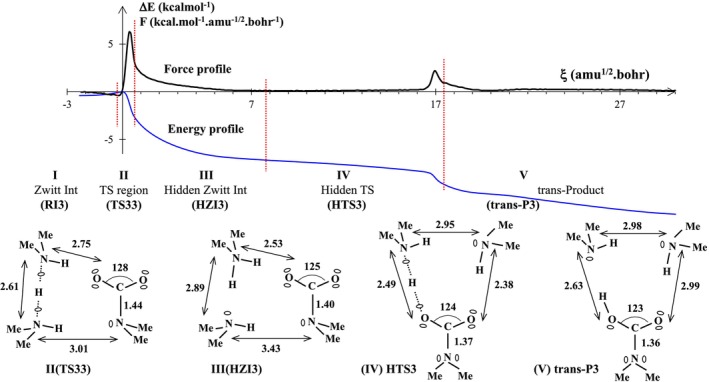
Energy and reaction force profiles, ELF topological regions, and the most relevant ELF bonding representation along the reaction path covering the third step of the reaction R3 connecting the reaction intermediate (RI3) to the final product (trans‐P3). Note that “Me” stands for methyl group. Bond lengths are in angstroms and ∠OCO angle in degrees.

Given the spatial configuration of the atoms within the RI3 reaction intermediate, we are tempted to think that two more successive proton transfers should lead to the final product formation, i.e., a carbamic acid forming two hydrogen bonds with the DMA dimer. Figure 7 shows some important properties of the third part of the reaction pathway of reaction R3: the energy profile and reaction force profile are illustrated on the top panel, and the topological domains and corresponding structures are shown on the bottom panel. There are 453 points along the IRC curve. Five topological domains of structural stability have been identified along the IRC path. Below the graph for each domain, a schematic representation of the ELF basins is depicted.

The second domain (labeled as II) corresponds to the transition state region, which covers a narrow range of reaction coordinates, from −0.5 to 0.7 amu^1/2^·Bohr. It is schematically represented by the optimized transition structure TS33. The key topological event within this transition domain is the proton transfer between two dimethylamine units. In other words, a roaming (delocalized) proton travels from one electronegative center to another by forming two attractors with the two nitrogen atoms and carrying a tiny negative charge (0.4 e).

**FIGURE 8:**
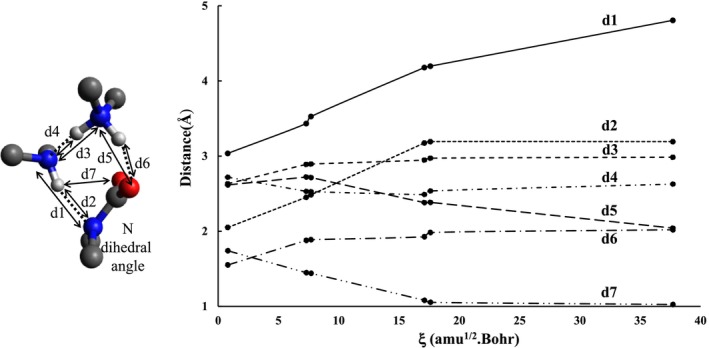
Some relevant geometrical parameters of the hidden structures (HZI3 and HTS3) vs. IRC.

Next come the two longest hidden domains that range from ξ ≅ 0.7 to 7.7 amu^1/2^·Bohr (running over 77 points, from the 35th to the 111th point) for domain III, and from 7.7 to 17.5 amu^1/2^·Bohr (running over 110 points, from the 112th to the 221st point) for domain IV. By definition, these two domains can only be represented by non‐optimized structures on the PES: hidden zwitterionic intermediate (HZI3) and hidden transition structure (HTS3). The last step of structural stability (labeled as V) corresponds to the domain of the final product, namely the carbamic acid in hydrogen bonding interaction with the DMA dimer. This domain runs over 232 points, from the 222nd point to the 453rd point. The representative topological structure for each of the two hidden domains clearly shows that it is a zwitterionic structure, facilitating both the detachment of (DMA)_2_H^+^ from the nitrogen center of the carbamate and the last proton transfer to complete the formation of the final product. We will examine the eight geometric parameters (seven distances d1‐d7 and one dihedral angle) plotted in Figure 8 to provide an overview of the geometric changes occurring over the last three domains of the reaction pathway.

To properly appreciate the geometric changes within each topological domain, we selected two points at the beginning and end of each domain (Figure 8). A close look at the results reported in Figure 8 shows that:

1. The distances d1 and d2 increase as the reaction progresses from domain III to IV, then to domain V. This means that the hydrogen bond between the DMA dimer and the carbamate nitrogen weakens with this distance and vanishes at the end of this reaction.
2. The d7 distance increasing over domain III starts to decrease within domain IV, reaching its minimum value (2.041 Å) at the end of domain V. This latter value is the geometric signature of the formation of a hydrogen bond between the H atom of DMA and the O atom of carbamate.
3. Two distances, d3‐d4, continuously increase when the intrinsic reaction coordinate (ξ) increases. At the end of the domain V, these distances are slightly smaller than their values in the pure DMA dimer (d3 = 3.158 Å and d4 = 2.193 Å).
4. The variations of two distances d5 and d6 directly exhibit the transfer of the 3rd hydrogen in the reaction R3. The distance d5, which decreases on domains III and IV, to reach its minimum value at the end of domain IV (2.489 A), increases slightly on domain V. At the same time, the distance d6 decreases steadily to reach its minimum value (1.025 Å) at the end of domain V, thus transforming the carbamate into carbamic acid.
5. The dihedral angle around the carbamate nitrogen (denoted as N‐dihedral) steadily decreases upon increasing the intrinsic reaction coordinate. The final value of this parameter, 176.3°, shows that the CNCC part of the carbamic acid is rather planar.

We underline that the transformation of RI3 (the only real species containing carbamate) into a trans‐product, involving carbamic acid, undergoes without difficulty because of a low activation energy that can be overcome by the energy of formation released. Despite our attempt, we failed to optimize a zwitterionic structure involving a carbamate anion. This is consistent with the topological description of structural stability domains, according to which such a zwitterionic structure is only a hidden structure on the IRC path. Consequently, the ultimate and unique product of the reaction is carbamic acid in non‐covalent interaction with a DMA dimer. The interaction energy of this complex was calculated to be 19 kcal/mol, which reflects the energy of two hydrogen bonds formed between two moieties. Our results are not only in excellent agreement with those of the recent joint experimental/theoretical study on CO_2_ conversion in pure dipropylamine liquid [10], they also provide a systematic and detailed analysis based on the ELF topology.

At this point, the question that naturally arises is whether the hidden domains identified throughout the third step of the reaction R3 could be transformed into two real domains when studying CO_2_ + (DMA)_n_ for *n* ≥ 4. Further research is underway on the reaction CO_2_ + (DMA)_n_ with *n* ≥ 4. Promising preliminary results, obtained for *n* = 4 and 5, indicate that the two hidden domains (Figure 7) disappear in favor of two real domains: a transition domain and an intermediate one. The latter is characterized by a zwitterionic complex composed of a carbamate interacting with a protonated DMA cluster.

## 4 Conclusions

A systematic theoretical study of the energetic/electronic features ruling the CO_2_ conversion reaction by a secondary amine, namely dimethylamine, has been investigated within the DFT framework and using the ELF topological approach. The main results of this work are summarized as follows:

1. The ultimate product of the CO_2_ + (DMA)_n_, with *n* = 1–3, is the carbamic acid when *n* = 1, and the carbamic acid in non‐covalent interaction with DMA mon‐ or dimer for *n* = 2–3.
2. The reaction R1 (CO_2_ + DMA‐monomer) occurs through two transition states. The ultimate product (carbamic acid) is obtained very slowly because of an activation barrier that is too high (ΔG^‡^ ≅ 38 kcal/mol).
3. The reaction R2 (CO_2_ + DMA‐dimer) occurs through three transition states. The rate‐limiting step has an activation energy not too large (ΔG^‡^ ≅ 11 kcal/mol) implying a rather high rate constant.
4. In contrast, the three‐step reaction R3 (CO_2_ + DMA‐trimer), the assistance of a third amine decreases the activation barrier for the rate‐limiting step (ΔG^‡^ ≅ 11 kcal/mol) and thus significantly increases the rate constant.
5. For all three reactions (R1, R2, and R3), the first species formed is a tetrel‐bonded complex that binds CO_2_ while making it slightly bent.

Thanks to the ELF topological analysis of the electronic structures along the IRC path, not only were we able to identify the different domains of structural stability (represented by stationary points), but we were also able to rationalize the more or less broad and rather flat shoulders into hidden domains, either as intermediate regions or as transition zones. We observed that the hidden domains become real domains (identifiable by a stationary point) along the IRC path when going from monomer to dimer and then to trimer of DMA interacting with CO_2_. Therefore, we must emphasize that the size of the DMA cluster is a parameter to control the CO_2_ conversion reaction.

## Supporting information


**Video S1.** The ELF cartoon of the bond breaking/forming processes along the reaction R1.

**Data S1.** Supporting Information.

## Data Availability

The data that support the findings of this study are available from the corresponding author upon reasonable request.

## References

[1] Y. Matsuzaki , H. Yamada , F. A. Chowdhury , T. Higashii , S. Kazama , and M. Onoda , “Ab Initio Study of CO_2_ Capture Mechanisms in Monoethanolamine Aqueous Solution: Reaction Pathways From Carbamate to Bicarbonate,” Energy Procedia 37 (2013): 400–406, 10.1016/j.egypro.2013.05.124. 24003832

[2] A. Raza , R. Gholami , R. Rezaee , V. Rasouli , and M. Rabiei , “Significant Aspects of Carbon Capture and Storage – A Review,” Petroleum 5 (2019): 335–340, 10.1016/j.petlm.2018.12.007.

[3] T. M. Gür , “Carbon Dioxide Emissions, Capture, Storage and Utilization: Review of Materials, Processes and Technologies,” Progress in Energy and Combustion Science 89 (2022): 100965, 10.1016/j.pecs.2021.100965.

[4] Q.‐W. Song , R. Ma , P. Liu , K. Zhang , and L.‐N. He , “Recent Progress in CO_2_ Conversion Into Organic Chemicals by Molecular Catalysis,” Green Chemistry 25 (2023): 6538–6560, 10.1039/D3GC01892J.

[5] R. J. Detz , C. J. Ferchaud , A. J. Kalkman , et al., “Electrochemical CO_2_ Conversion Technologies: State‐Of‐The‐Art and Future Perspectives,” Sustainable Energy & Fuels 7, no. 23 (2023): 545–5472, 10.1039/D3SE00775H.

[6] B. A. Jackson , S. G. Dale , M. Camarasa‐Gómez , and E. Miliordos , “Introducing Novel Materials With Diffuse Electrons for Applications in Redox Catalysis and Quantum Computing via Theoretical Calculations,” Journal of Physical Chemistry C 127 (2023): 9295–9308, 10.1021/acs.jpcc.3c00675.

[7] H. Huang , L. Xue , and Y. Bu , “Multifunctional Roles of Clathrate Hydrate Nanoreactors for CO_2_ Reduction,” Chemistry – A European Journal 29 (2023): e202302253, 10.1002/chem.202302253. 37580312

[8] Y. Meng , H. Huang , Y. Zhang , Y. Cao , H. Lu , and X. Li , “Recent Advances in the Theoretical Studies on the Electrocatalytic CO_2_ Reduction Based on Single and Double Atoms,” Frontiers in Chemistry 11 (2023): 1172146, 10.3389/fchem.2023.1172146. 37056353 PMC10086683

[9] R. E. Siegel , S. Pattanayak , and L. A. Berben , “Reactive Capture of CO_2_: Opportunities and Challenges,” ACS Catalysis 13 (2023): 766–784, 10.1021/acscatal.2c05019.

[10] G. Manca , F. Barzagli , J. Nagy , M. Munzarová , M. Peruzzini , and A. Ienco , “Unraveling the Mechanism and the Role of Hydrogen Bonds in CO_2_ Capture by Diluent‐Free Amine Sorbents Through a Combination of Experimental and Theoretical Methods,” Fuel 378 (2024): 132859, 10.1016/j.fuel.2024.132859.

[11] B. Mondal , J. Song , F. Neese , and S. Ye , “Bio‐Inspired Mechanistic Insights Into CO_2_ Reduction,” Current Opinion in Chemical Biology 25 (2015): 103–109, 10.1016/j.cbpa.2014.12.022. 25588961

[12] A. Álvarez , M. Borges , J. J. Corral‐Pérez , et al., “CO_2_ Activation Over Catalytic Surfaces,” ChemPhysChem 18 (2017): 3135–3141, 10.1002/cphc.201700782. 28851111

[13] P. Sreejyothi and S. K. Mandal , “From CO_2_ Activation to Catalytic Reduction: A Metal‐Free Approach,” Chemical Science (2020): 10571–10593, 10.1039/D0SC03528A. 34094313 PMC8162374

[14] A. Yanagimachi , K. Koyasu , D. Y. Valdivielso , et al., “Size‐Specific, Dissociative Activation of Carbon Dioxide by Cobalt Cluster Anions,” Journal of Physical Chemistry C 120 (2016): 14209–14215, 10.1021/acs.jpcc.6b04360.

[15] G. Liu , S. M. Ciborowski , Z. Zhu , Y. Chen , X. Zhang , and K. H. Bowen , “The Metallo‐Formate Anions, M(CO_2_)−, M = Ni, pd, Pt, Formed by Electron‐Induced CO_2_ Activation,” Physical Chemistry Chemical Physics 21 (2019): 10955–10960, 10.1039/C9CP01915D. 31099819

[16] C. Vogt , M. Monai , E. B. Sterk , et al., “Understanding Carbon Dioxide Activation and Carbon–Carbon Coupling Over Nickel,” Nature Communications 10 (2019): 5330, 10.1038/s41467-019-12858-3. PMC6877608 31767838

[17] Z. Yue , C. Ou , N. Ding , L. Tao , J. Zhao , and J. Chen , “Advances in Metal Phthalocyanine Based Carbon Composites for Electrocatalytic CO_2_ Reduction,” ChemCatChem 12 (2020): 6948–6955, 10.1002/cctc.202001126.

[18] X.‐Y. He , Y. Z. Liu , J. J. Chen , X. Lan , X. N. Li , and S. G. He , “Size‐Dependent Reactivity of con– (*n* = 5–25) Cluster Anions Toward Carbon Dioxide,” Journal of Physical Chemistry Letters 14 (2023): 6948–6955, 10.1021/acs.jpclett.3c01478. 37498356

[19] D. Niks and R. Hille , “Reductive Activation of CO_2_ by Formate Dehydrogenases,” in Methods in Enzymology, vol. 613 (Elsevier, 2018), 277–295. 30509470 10.1016/bs.mie.2018.10.013

[20] S. Chandra Sau , R. Bhattacharjee , P. K. Hota , et al., “Transforming Atmospheric CO_2_ Into Alternative Fuels: A Metal‐Free Approach Under Ambient Conditions,” Chemical Science 10 (2019): 1879–1884, 10.1039/C8SC03581D. 30842857 PMC6371756

[21] H. Kayi , R. I. Kaiser , and J. D. Head , “A Computational Study on the Structures of Methylamine–Carbon Dioxide–Water Clusters: Evidence for the Barrier Free Formation of the Methylcarbamic Acid Zwitterion (CH3NH2+COO−) in Interstellar Water Ices,” Physical Chemistry Chemical Physics 13 (2011): 11083–11098, 10.1039/c0cp01962c. 21311787

[22] S. A. Didas , M. A. Sakwa‐Novak , G. S. Foo , C. Sievers , and C. W. Jones , “Effect of Amine Surface Coverage on the co‐Adsorption of CO_2_ and Water: Spectral Deconvolution of Adsorbed Species,” Journal of Physical Chemistry Letters 5 (2014): 4194–4200, 10.1021/jz502032c. 26278953

[23] S. A. Didas , R. Zhu , N. A. Brunelli , D. S. Sholl , and C. W. Jones , “Thermal, Oxidative and CO_2_ Induced Degradation of Primary Amines Used for CO_2_ Capture: Effect of Alkyl Linker on Stability,” Journal of Physical Chemistry C 118 (2014): 12302–12311, 10.1021/jp5025137.

[24] P.‐H. Lin and D. S. H. Wong , “Carbon Dioxide Capture and Regeneration With Amine/Alcohol/Water Blends,” International Journal of Greenhouse Gas Control 26 (2014): 69–75, 10.1016/j.ijggc.2014.04.020.

[25] C. Ma , F. Pietrucci , and W. Andreoni , “CO_2_ Capture and Release in Amine Solutions: To What Extent Can Molecular Simulations Help Understand the Trends?,” Molecules 28 (2023): 6447, 10.3390/molecules28186447. 37764223 PMC10534568

[26] B. Yoon and G. A. Voth , “Elucidating the Molecular Mechanism of CO_2_ Capture by Amino Acid Ionic Liquids,” Journal of the American Chemical Society 145 (2023): 15663–15667, 10.1021/jacs.3c03613. 37439824 PMC10375530

[27] R. B. Said , J. M. Kolle , K. Essalah , B. Tangour , and A. Sayari , “A Unified Approach to CO_2_–Amine Reaction Mechanisms,” ACS Omega 5 (2020): 26125–26133, 10.1021/acsomega.0c03727. 33073140 PMC7557993

[28] R. B. Said , S. Rahali , C. Yan , M. Seydou , B. Tangour , and A. Sayari , “CO_2_ Capture by Diamines in Dry and Humid Conditions: A Theoretical Approach,” Journal of Physical Chemistry A 127 (2023): 7756–7763, 10.1021/acs.jpca.3c04416. 37698444

[29] C. Mealli , R. Hoffmann , and A. Stockis , “Molecular Orbital Analysis of the Bonding Capabilities of Carbon Disulfide and Carbon Dioxide Toward Transition Metal Fragments,” Inorganic Chemistry 23 (1984): 56–65, 10.1021/ic00169a014.

[30] R. J. Ellis , “Tackling unintelligent design,” Nature 463 (2010): 164–165, 10.1038/463164a. 20075906

[31] R. Zhang , X. Zhang , Q. Yang , H. Yu , Z. Liang , and X. Luo , “Analysis of the Reduction of Energy Cost by Using MEA‐MDEA‐PZ Solvent for Post‐Combustion Carbon Dioxide Capture (PCC),” Applied Energy 205 (2017): 1002–1011, 10.1016/j.apenergy.2017.08.130.

[32] B. Yu , H. Yu , K. Li , et al., “Characterisation and Kinetic Study of Carbon Dioxide Absorption by an Aqueous Diamine Solution,” Applied Energy 208 (2017): 1308–1317, 10.1016/j.apenergy.2017.09.023.

[33] S. Singto , T. Supap , R. Idem , et al., “Synthesis of New Amines for Enhanced Carbon Dioxide (CO_2_) Capture Performance: The Effect of Chemical Structure on Equilibrium Solubility, Cyclic Capacity, Kinetics of Absorption and Regeneration, and Heats of Absorption and Regeneration,” Separation and Purification Technology 167 (2016): 97–107, 10.1016/j.seppur.2016.05.002.

[34] L. Li and G. Rochelle , “CO_2_ Mass Transfer and Solubility in Aqueous Primary and Secondary Amine,” Energy Procedia 63 (2014): 1487–1496, 10.1016/j.egypro.2014.11.158.

[35] N. El Hadri , D. V. Quang , E. L. V. Goetheer , et al., “Aqueous amine solution characterization for post‐combustion CO_2_ capture process,” Applied Energy 185 (2017): 1433–1449, 10.1016/j.apenergy.2016.03.043.

[36] W. Conway , S. Bruggink , Y. Beyad , et al., “CO_2_ Absorption Into Aqueous Amine Blended Solutions Containing Monoethanolamine (MEA), N,N‐Dimethylethanolamine (DMEA), N,N‐Diethylethanolamine (DEEA) and 2‐Amino‐2‐Methyl‐1‐Propanol (AMP) for Post‐Combustion Capture Processes,” Chemical Engineering Science 126 (2015): 446–454, 10.1016/j.ces.2014.12.053.

[37] F. Barzagli , C. Giorgi , F. Mani , and M. Peruzzini , “Reversible Carbon Dioxide Capture by Aqueous and Non‐Aqueous Amine‐Based Absorbents: A Comparative Analysis Carried out by 13C NMR Spectroscopy,” Applied Energy 220 (2018): 208–219, 10.1016/j.apenergy.2018.03.076.

[38] J. K. Mannisto , L. Pavlovic , T. Tiainen , et al., “Mechanistic Insights Into Carbamate Formation From CO_2_ and Amines: The Role of Guanidine–CO_2_ Adducts,” Catalysis Science & Technology 11, no. 20 (2021): 6877–6886, 10.1039/D1CY01433A.

[39] M. Ferrer , J. Elguero , I. Alkorta , and L. M. Azofra , “Understanding the Coupling of Non‐Metallic Heteroatoms to CO_2_ From a Conceptual DFT Perspective,” Journal of Molecular Modeling 30 (2024): 201, 10.1007/s00894-024-05992-3. 38853233 PMC11162977

[40] P. D. Holtom , C. J. Bennett , Y. Osamura , N. J. Mason , and R. I. Kaiser , “A Combined Experimental and Theoretical Study on the Formation of the Amino Acid Glycine (NH2 CH2 COOH) and Its Isomer (CH3 NHCOOH) in Extraterrestrial Ices,” Astrophysical Journal 626, no. 2 (2005): 940–952, 10.1086/430106.

[41] S. Li , H. G. Kjaergaard , and L. du , “Infrared Spectroscopic Probing of Dimethylamine Clusters in an Ar Matrix,” Journal of Environmental Sciences 40 (2016): 51–59, 10.1016/j.jes.2015.09.012. 26969545

[42] M. J. Tubergen and R. L. Kuczkowski , “Microwave Spectrum and Structure of the Dimethylamine Dimer: Evidence for a Cyclic Structure,” Journal of Chemical Physics 100 (1994): 3377–3383, 10.1063/1.466381.

[43] E. M. Cabaleiro‐Lago and M. A. Ríos , “An Ab Initio Study of the Interaction in Dimethylamine Dimer and Trimer,” Journal of Chemical Physics 113 (2000): 9523–9531, 10.1063/1.1321314.

[44] M. J. Frisch , “Gaussian 09 Revision D.01,” 2013.

[45] T. Körzdörfer , J. S. Sears , C. Sutton , et al., “Long‐Range Corrected Hybrid Functionals for π‐Conjugated Systems: Dependence of the Range‐Separation Parameter on Conjugation Length,” Journal of Chemical Physics 135 (2011): 204107, 10.1063/1.3663856. 22128928

[46] J.‐D. Chai and M. Head‐Gordon , “Systematic Optimization of Long‐Range Corrected Hybrid Density Functionals,” Journal of Chemical Physics 128 (2008): 084106, 10.1063/1.2834918. 18315032

[47] A. D. McLean and G. S. Chandler , “Contracted Gaussian Basis Sets for Molecular Calculations. I. Second Row Atoms, Z =11–18,” Journal of Chemical Physics 72 (1980): 5639–5648, 10.1063/1.438980.

[48] R. Krishnan , J. S. Binkley , R. Seeger , and J. A. Pople , “Self‐Consistent Molecular Orbital Methods. XX. A Basis Set for Correlated Wave Functions,” Journal of Chemical Physics 72 (1980): 650–654, 10.1063/1.438955.

[49] G. Santra , R. Calinsky , and J. M. L. Martin , “Benefits of Range‐Separated Hybrid and Double‐Hybrid Functionals for a Large and Diverse Data Set of Reaction Energies and Barrier Heights,” Journal of Physical Chemistry A 126 (2022): 5492–5505, 10.1021/acs.jpca.2c03922. 35930677 PMC9393870

[50] K. Fukui , “Formulation of the Reaction Coordinate,” Journal of Physical Chemistry 74 (1970): 4161–4163, 10.1021/j100717a029.

[51] M. Page and J. W. McIver , “On Evaluating the Reaction Path Hamiltonian,” Journal of Chemical Physics 88 (1988): 922–935, 10.1063/1.454172.

[52] M. Page , C. Doubleday , and J. W. McIver, Jr. , “Following Steepest Descent Reaction Paths. The Use of Higher Energy Derivatives With a b i n i t i o Electronic Structure Methods,” Journal of Chemical Physics 93 (1990): 5634–5642, 10.1063/1.459634.

[53] F. Weinhold and C. R. Landis , Discovering Chemistry With Natural Bond Orbitals (Wiley, 2012).

[54] A. Toro‐Labbé , “Characterization of Chemical Reactions From the Profiles of Energy, Chemical Potential, and Hardness,” Journal of Physical Chemistry A 103 (1999): 4398–4403, 10.1021/jp984187g.

[55] C. Barrales‐Martínez and P. Jaque , “A Deeper Analysis of the Role of Synchronicity on the Bell–Evans–Polanyi Plot in Multibond Chemical Reactions: A Path‐Dependent Reaction Force Constant,” Physical Chemistry Chemical Physics 24 (2022): 14772–14779, 10.1039/D2CP01460B. 35686531

[56] A. D. Becke and K. E. Edgecombe , “A Simple Measure of Electron Localization in Atomic and Molecular Systems,” Journal of Chemical Physics 92 (1990): 5397–5403, 10.1063/1.458517.

[57] B. Silvi and A. Savin , “Classification of Chemical Bonds Based on Topological Analysis of Electron Localization Functions,” Nature 371 (1994): 683–686, 10.1038/371683a0.

[58] M. E. Alikhani , F. Fuster , and B. Silvi , “What Can Tell the Topological Analysis of ELF on Hydrogen Bonding?,” Structural Chemistry 16 (2005): 203–210, 10.1007/s11224-005-4451-z.

[59] S. Noury , X. Krokidis , F. Fuster , and B. Silvi , “Computational Tools for the Electron Localization Function Topological Analysis,” Computers & Chemistry 23 (1999): 597–604, 10.1016/S0097-8485(99)00039-X.

[60] V. Polo , J. Andres , R. Castillo , S. Berski , and B. Silvi , “Understanding the Molecular Mechanism of the 1,3‐Dipolar Cycloaddition Between Fulminic Acid and Acetylene in Terms of the Electron Localization Function and Catastrophe Theory,” Chemistry – A European Journal 10, no. 20 (2004): 5165–5172, 10.1002/chem.200400161. 15372667

[61] A. Ćmikiewicz , A. J. Gordon , and S. Berski , “Characterisation of the Reaction Mechanism Between Ammonia and Formaldehyde From the Topological Analysis of ELF and Catastrophe Theory Perspective,” Structural Chemistry 29 (2018): 243–255, 10.1007/s11224-017-1024-x.

[62] J. Andrés , P. González‐Navarrete , V. S. Safont , and B. Silvi , “Curly Arrows, Electron Flow, and Reaction Mechanisms From the Perspective of the Bonding Evolution Theory,” Physical Chemistry Chemical Physics 19 (2017): 29031–29046, 10.1039/C7CP06108K. 29077108

[63] L. R. Domingo , M. J. Aurell , P. Pérez , and J. A. Sáez , “Understanding the Origin of the Asynchronicity in Bond‐Formation in Polar Cycloaddition Reactions. A DFT Study of the 1,3‐Dipolar Cycloaddition Reaction of Carbonyl Ylides With 1,2‐Benzoquinones,” RSC Advances 2 (2012): 1334–1342, 10.1039/C1RA00717C.

[64] B. Arstad , R. Blom , and O. Swang , “CO_2_ Absorption in Aqueous Solutions of Alkanolamines: Mechanistic Insight From Quantum Chemical Calculations,” Journal of Physical Chemistry A 111 (2007): 1222–1228, 10.1021/jp065301v. 17266286

[65] M. W. Hahn , J. Jelic , E. Berger , K. Reuter , A. Jentys , and J. A. Lercher , “Role of Amine Functionality for CO_2_ Chemisorption on Silica,” Journal of Physical Chemistry B 120 (2016): 1988–1995, 10.1021/acs.jpcb.5b10012. 26700549

[66] W. Buijs and S. De Flart , “Direct Air Capture of CO_2_ With an Amine Resin: A Molecular Modeling Study of the CO_2_ Capturing Process,” Industrial & Engineering Chemistry Research 56 (2017): 12297–12304, 10.1021/acs.iecr.7b02613. 29142339 PMC5678289

[67] D. S. Mebane , J. D. Kress , C. B. Storlie , D. J. Fauth , M. M. L. Gray , and K. Li , “Transport, Zwitterions, and the Role of Water for CO_2_ Adsorption in Mesoporous Silica‐Supported Amine Sorbents,” Journal of Physical Chemistry C 117 (2013): 26617–26627, 10.1021/jp4076417.

[68] A. C. Legon , “Tetrel, Pnictogen and Chalcogen Bonds Identified in the Gas Phase Before They Had Names: A Systematic Look at Non‐Covalent Interactions,” Physical Chemistry Chemical Physics 19 (2017): 14884–14896, 10.1039/C7CP02518A. 28561824

[69] I. Alkorta , J. Elguero , and J. E. Del Bene , “Azines as Electron‐Pair Donors to CO_2_ for N···C Tetrel Bonds,” Journal of Physical Chemistry A 121 (2017): 8017–8025, 10.1021/acs.jpca.7b08505. 28945437

[70] T. Lu , J. Zhang , Q. Gou , and G. Feng , “Structure and C⋯N Tetrel‐Bonding of the Isopropylamine–CO_2_ Complex Studied by Microwave Spectroscopy and Theoretical Calculations,” Physical Chemistry Chemical Physics 22 (2020): 8467–8475, 10.1039/D0CP00925C. 32285060

[71] F. De Vleeschouwer , F. De Proft , Ö. Ergün , et al., “A Combined Experimental/Quantum‐Chemical Study of Tetrel, Pnictogen, and Chalcogen Bonds of Linear Triatomic Molecules,” Molecules 26 (2021): 6767, 10.3390/molecules26226767. 34833858 PMC8623034

[72] S. Chandra , N. Mahapatra , N. Ramanathan , and K. Sundararajan , “CO_2_‐NH_3_ Dimers: Dominance of π‐Hole Driven Tetrel Bonding Over Hydrogen Bonding,” Chemical Physics Letters 828 (2023): 140722, 10.1016/j.cplett.2023.140722.

[73] P. Politzer , J. S. Murray , and T. Clark , “Explicit Inclusion of Polarizing Electric Fields in σ‐ and π‐Hole Interactions,” Journal of Physical Chemistry A 123 (2019): 10123–10130, 10.1021/acs.jpca.9b08750. 31647237

[74] P. Politzer and J. S. Murray , “Electrostatics and Polarization in σ‐ and π‐Hole Noncovalent Interactions: An Overview,” ChemPhysChem 21 (2020): 579–588, 10.1002/cphc.201900968. 31733136

[75] L. M. Azofra , I. Alkorta , J. Elguero , and A. Toro‐Labbé , “Mechanisms of Formation of Hemiacetals: Intrinsic Reactivity Analysis,” Journal of Physical Chemistry A 116 (2012): 8250–8259, 10.1021/jp304495f. 22784613

[76] C. Trujillo , G. Sánchez‐Sanz , I. Alkorta , and J. Elguero , “Computational Study of Proton Transfer in Tautomers of 3‐ and 5‐Hydroxypyrazole Assisted by Water,” ChemPhysChem 16 (2015): 2140–2150, 10.1002/cphc.201500317. 26033797

[77] H. Joo , E. Kraka , W. Quapp , and D. Cremer , “The Mechanism of a Barrierless Reaction: Hidden Transition State and Hidden Intermediates in the Reaction of Methylene With Ethene,” Molecular Physics 105 (2007): 2697–2717, 10.1080/00268970701620677.

[78] E. Kraka and D. Cremer , “Computational Analysis of the Mechanism of Chemical Reactions in Terms of Reaction Phases: Hidden Intermediates and Hidden Transition States,” Accounts of Chemical Research 43 (2010): 591–601, 10.1021/ar900013p. 20232791

[79] T. Sexton , E. Kraka , and D. Cremer , “Extraordinary Mechanism of the Diels–Alder Reaction: Investigation of Stereochemistry, Charge Transfer, Charge Polarization, and Biradicaloid Formation,” Journal of Physical Chemistry A 120 (2016): 1097–1111, 10.1021/acs.jpca.5b11493. 26785172

[80] H. S. Rzepa and C. Wentrup , “Mechanistic Diversity in Thermal Fragmentation Reactions: A Computational Exploration of CO and CO 2 Extrusions From Five‐Membered Rings,” Journal of Organic Chemistry 78 (2013): 7565–7574, 10.1021/jo401146k. 23795601

[81] A. Armstrong , R. A. Boto , P. Dingwall , et al., “The Houk–List Transition States for Organocatalytic Mechanisms Revisited,” Chemical Science 5 (2014): 2057–2071, 10.1039/C3SC53416B.

[82] D. Roca‐López , V. Polo , T. Tejero , and P. Merino , “Understanding Bond Formation in Polar One‐Step Reactions. Topological Analyses of the Reaction Between Nitrones and Lithium Ynolates,” Journal of Organic Chemistry 80 (2015): 4076–4083, 10.1021/acs.joc.5b00413. 25803829

[83] R. Durán , C. Barrales‐Martínez , and R. A. Matute , “Hidden Intermediate Activation: A Concept to Elucidate the Reaction Mechanism of the Schmittel Cyclization of Enyne–Allenes,” Physical Chemistry Chemical Physics 25 (2023): 6050–6059, 10.1039/D2CP04684A. 36458512

[84] V. Labet , A. Geoffroy‐Neveux , and M. E. Alikhani , “How to Search for and Reveal a Hidden Intermediate? The ELF Topological Description of Non‐Synchronicity in Double Proton Transfer Reactions Under Oriented External Electric Field,” Journal of Molecular Modeling 30 (2024): 367, 10.1007/s00894-024-06163-0. 39365459

[85] X. Krokidis , R. Vuilleumier , D. Borgis , et al., “A Topological Analysis of the Proton Transfer in H5O+2,” Molecular Physics 96 (1999): 265–273, 10.1080/00268979909482959.

[86] J. M. Bowman , “Skirting the Transition State, a New Paradigm in Reaction Rate Theory,” National Academy of Sciences of the United States of America 103 (2006): 16061–16062, 10.1073/pnas.0607810103. PMC1637535 17060637

[87] X. Krokidis , V. Goncalves , A. Savin , and B. Silvi , “How Malonaldehyde Bonds Change During Proton Transfer,” Journal of Physical Chemistry A 102 (1998): 5065–5073, 10.1021/jp9734282.

[88] A. Geoffroy‐Neveux , V. Labet , and M. E. Alikhani , “Influence of an Oriented External Electric Field on the Mechanism of Double Proton Transfer Between Pyrazole and Guanidine: From an Asynchronous Plateau Transition State to a Synchronous or Stepwise Mechanism,” Journal of Physical Chemistry A 126 (2022): 3057–3071, 10.1021/acs.jpca.1c10553. 35544749

[89] B. Silvi , “The Synaptic Order: A Key Concept to Understand Multicenter Bonding,” Journal of Molecular Structure 614 (2002): 3–10, 10.1016/S0022-2860(02)00231-4.

[90] C. Lepetit , V. Maraval , Y. Canac , and R. Chauvin , “On the Nature of the Dative Bond: Coordination to Metals and Beyond. The Carbon Case,” Coordination Chemistry Reviews 308 (2016): 59–75, 10.1016/j.ccr.2015.07.018.

[91] P. Salvador , E. Vos , I. Corral , and D. M. Andrada , “Beyond the Classical Electron‐Sharing and Dative Bond Picture: Case of the Spin‐Polarized Bond,” Angewandte Chemie International Edition 60 (2021): 1498–1502, 10.1002/anie.202010948. 32866305 PMC7839703

[92] J. E. Carpenter , “NBO 6.0,” 2013.

