# Supplementary material for: Modulation of the Product Upon the Reaction of CO2 With Dimethylamine Cluster: A Topological Analysis of the Reaction Mechanism
Source: J Comput Chem. 2025 May 14;46(13):e70135. doi: 10.1002/jcc.70135 (PMC12078888; doi:10.1002/jcc.70135)
Supplement: Supplementary file 2 — Data S1. Supporting Information. [file JCC-46-0-s001.docx]

Three dimethylamine molecules transform a CO_2_ into a carbamic acid: a topological analysis of the reaction mechanism.

Mohammad Esmaïl Alikhani ^1^ and Bernard Silvi ^2^

1. Sorbonne Université, MONARIS, CNRS-UMR 8233, F-75005 Paris, France.

E-mail: [esmail.alikhani@sorbonne-universite.fr](mailto:esmail.alikhani@sorbonne-universite.fr) ID: <https://orcid.org/0000-0001-8412-414>7

1. Sorbonne Université, LCT, CNRS-UMR 7616, F-75005 Paris, France.

E-mail: sbernard@lct.jussieu.fr ID: https://orcid.org/0000-0002-3872-0121

| **SI-1: Energetic diagram for the reactions R1 and R2**  **SI-2: Optimized geometries of CO_2_-DMA, CO_2_-(DMA)_2_, and CO_2_-(DMA)_3_**  **SI-3: Energetic diagram *vs.* dipole moment for the reaction R1**  **SI-4: Energetic diagram for the second step of the reaction R2 using MP2 method**  **SI-5: Cartesian coordinates of stationary points at MP2/6-311++G(2d,2p) level**  **SI-6: Cartesian coordinates of stationary points at wB97XD/6-311++G(2d,2p) level** | **2**  **3**  **5**  **6**  **7**  **9** |
| --- | --- |

**SI-1: Energetic diagram for the reactions R1 and R2**

Relative energy of minima and transition states found for the reaction R1. Relative electronic energy (ΔE_el_), electronic energy corrected for the zero-point energy (ΔE_el_^ZPE^), enthalpy (ΔH^°^), and free energy (ΔG^°^) are reported for the wB97XD and MP2 methods.

Relative energy of minima and transition states found for the reaction R2. Relative electronic energy (ΔE_el_), electronic energy corrected for the zero-point energy (ΔE_el_^ZPE^), enthalpy (ΔH^°^), and free energy (ΔG^°^) are reported for the wB97XD functional.

**SI-2: Optimized geometries of CO_2_-DMA, CO_2_-(DMA)_2_, and CO_2_-(DMA)_3_**

**Optimized geometries of the CO_2_ + DMA reaction**

**Optimized geometries of the CO_2_ + (DMA)_2_ reaction**

**Optimized geometries of the CO_2_ + (DMA)_3_ reaction**

**SI-3: Energetic diagram *vs.* dipole moment for the reaction R1**


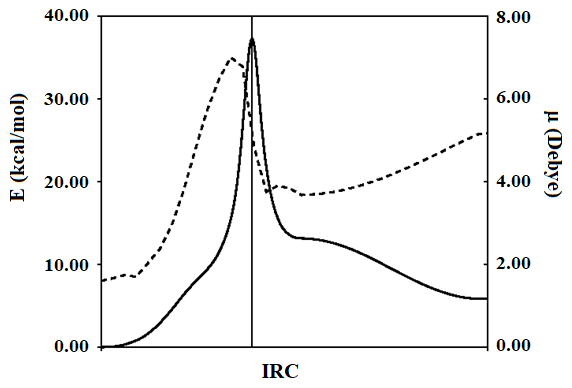


*CO2 + DMA complex Relative energy (full line) and dipole moment (dashed line) profiles along the IRC*

**SI-4: Energetic diagram for the second step of the reaction R2**

Energy and reaction force profiles along the reaction path for the second step of the reaction R2 connecting the dative-bonded complex (Dat2) to the cis-product.

**SI-5: Cartesian coordinates of stationary points at MP2/6-311++G(2d,2p) level**

**CO_2_ MP2 = -188.2458098**

6 0 0.000000 0.000000 0.000000

8 0 0.000000 0.000000 1.169572

8 0 0.000000 0.000000 -1.169572

**DMA MP2 = -134.8151189**

7 0 -0.027310 0.594032 0.000000

1 0 0.791800 1.184323 0.000000

6 0 -0.027310 -0.225524 1.206234

6 0 -0.027310 -0.225524 -1.206234

1 0 0.788412 -0.957324 1.238363

1 0 0.041439 0.410882 2.083508

1 0 -0.966306 -0.771687 1.261189

1 0 0.788412 -0.957324 -1.238363

1 0 -0.966306 -0.771687 -1.261189

1 0 0.041439 0.410882 -2.083508

**DMA-CO_2_ TtB1 MP2 = -323.0676664**

6 0 1.581018 1.206003 -0.093223

7 0 1.062967 0.000023 0.545748

6 0 -1.701947 0.000052 -0.110213

8 0 -2.096841 0.000088 0.990332

8 0 -1.369617 0.000016 -1.232993

6 0 1.580681 -1.206132 -0.093164

1 0 1.324424 0.000001 1.521358

1 0 1.195148 1.255464 -1.108998

1 0 1.234655 2.084764 0.443441

1 0 2.674813 1.235379 -0.144923

1 0 2.674459 -1.235668 -0.145142

1 0 1.234343 -2.084752 0.443746

1 0 1.194545 -1.255718 -1.108832

**DMA-CO_2_ TS11 MP2 = -323.0053787 w(TS) = 1681i**

6 0 1.261238 1.219989 -0.059175

7 0 0.565420 0.000000 0.361540

6 0 1.261236 -1.219990 -0.059176

6 0 -0.909307 0.000001 -0.107325

8 0 -1.265954 0.000001 -1.255288

8 0 -1.480746 0.000000 1.057388

1 0 0.710915 2.085671 0.295675

1 0 2.260708 1.228458 0.365450

1 0 1.323133 1.253324 -1.146111

1 0 2.260706 -1.228461 0.365449

1 0 0.710912 -2.085671 0.295673

1 0 1.323132 -1.253324 -1.146112

1 0 -0.252849 0.000000 1.376449

**DMA-CO_2_ cis-P1 MP2 = -323.0532994**

6 0 1.415541 -1.152786 0.004666

7 0 0.568505 0.020130 -0.159755

6 0 1.194987 1.311309 0.069311

6 0 -0.786634 -0.174105 -0.020931

8 0 -1.319095 -1.259006 0.058931

8 0 -1.549565 0.961117 -0.001351

1 0 0.858312 -2.028017 -0.301383

1 0 2.297193 -1.045014 -0.620615

1 0 1.727231 -1.280453 1.042021

1 0 2.249677 1.223243 -0.168566

1 0 0.794170 2.075879 -0.594914

1 0 1.105359 1.647101 1.103800

1 0 -1.005562 1.742962 -0.120982

**DMA-CO_2_ TS12 MP2 = -323.0489085 w(TS) = 432i**

6 0 1.459330 -1.107201 -0.058646

7 0 0.561914 0.034886 -0.060470

6 0 -0.777487 -0.194224 0.010926

8 0 -1.545482 0.969281 0.035674

6 0 1.190152 1.336025 0.078170

8 0 -1.289888 -1.291445 0.079652

1 0 0.903724 -1.994687 -0.331502

1 0 2.257994 -0.936669 -0.777024

1 0 1.900094 -1.251746 0.927558

1 0 1.760881 1.581008 -0.816603

1 0 0.440240 2.094964 0.248522

1 0 1.873706 1.322916 0.926441

1 0 -1.619054 1.309725 -0.859411

**DMA-CO_2_ trans-P1 MP2 = -323.0662127**

6 0 1.441098 -1.142911 0.015065

7 0 0.579274 0.020431 -0.072023

6 0 -0.762048 -0.159569 -0.011463

8 0 -1.442815 1.030323 0.006639

6 0 1.223309 1.318023 0.016905

8 0 -1.340453 -1.234377 0.012199

1 0 0.838967 -2.034553 -0.100679

1 0 2.190112 -1.102592 -0.772812

1 0 1.949247 -1.174031 0.978856

1 0 -2.369591 0.764678 0.023023

1 0 2.052778 1.348732 -0.686150

1 0 0.521982 2.101763 -0.231766

1 0 1.613578 1.492153 1.019946

**SI-6: Cartesian coordinates of stationary points at wB97XD/6-311++G(2d,2p) level**

**CO_2_ HF = -188.5851466**

6 0 0.000000 0.000000 0.000000

8 0 0.000000 0.000000 1.156247

8 0 0.000000 0.000000 -1.156247

**DMA HF = -135.1668356**

7 0 -0.026624 0.581443 0.000000

1 0 0.773032 1.197485 0.000000

6 0 -0.026624 -0.221741 1.207969

6 0 -0.026624 -0.221741 -1.207969

1 0 0.796755 -0.950699 1.254654

1 0 0.033565 0.425175 2.082599

1 0 -0.963906 -0.777824 1.271993

1 0 0.796755 -0.950699 -1.254654

1 0 -0.963906 -0.777824 -1.271993

1 0 0.033565 0.425175 -2.082599

**DMA dimer HF = -270.3424622**

7 0 -1.582213 0.256816 0.520235

6 0 -2.119421 -1.020456 0.081680

6 0 -1.453193 1.211247 -0.568280

7 0 1.537376 -0.234461 0.563785

6 0 2.133278 1.010840 0.126351

6 0 1.608467 -1.266170 -0.449323

1 0 0.565445 -0.076818 0.810009

1 0 -2.167760 0.642962 1.246510

1 0 3.207476 0.876377 -0.019970

1 0 1.716901 1.393604 -0.820514

1 0 1.996449 1.775140 0.891261

1 0 2.652170 -1.520972 -0.647142

1 0 1.108696 -2.167499 -0.094225

1 0 1.153270 -0.974820 -1.411583

1 0 -1.417556 -1.481607 -0.615327

1 0 -2.228631 -1.688562 0.935004

1 0 -3.090628 -0.935519 -0.425858

1 0 -0.720050 0.837329 -1.285852

1 0 -2.393209 1.389857 -1.108821

1 0 -1.083500 2.161279 -0.184198

**DMA trimer HF = -405.524075**

7 0 0.397180 -1.741397 -0.118451

7 0 1.312492 1.218187 -0.122216

7 0 -1.706886 0.529411 -0.117011

1 0 -0.469318 -1.214865 -0.216176

1 0 -0.815896 1.014243 -0.212080

1 0 1.287607 0.206681 -0.242001

6 0 -2.626642 0.973359 -1.147173

6 0 -2.225718 0.736744 1.222046

6 0 2.181208 1.818624 -1.115665

6 0 1.722215 1.522678 1.235619

6 0 0.478665 -2.759053 -1.148820

6 0 0.467995 -2.296493 1.219777

1 0 -2.943156 2.020156 -1.027208

1 0 -2.160340 0.867511 -2.126436

1 0 -3.525697 0.353101 -1.136575

1 0 -2.517911 1.778388 1.420761

1 0 -3.104723 0.108891 1.383421

1 0 -1.472500 0.448636 1.956084

1 0 -0.279496 -3.549273 -1.042175

1 0 0.358116 -2.298950 -2.129178

1 0 1.460526 -3.237017 -1.122999

1 0 -0.289639 -3.070107 1.414000

1 0 1.450395 -2.744774 1.384448

1 0 0.338465 -1.501227 1.954146

1 0 2.764697 1.245285 1.450826

1 0 1.617984 2.593374 1.424400

1 0 1.078617 0.994508 1.939410

1 0 3.241589 1.556433 -0.983234

1 0 1.873261 1.503570 -2.112476

1 0 2.101578 2.906874 -1.067915

**DMA-CO_2_ TtB1 HF = -323.7582664**

6 0 1.562652 -1.216417 0.333311

7 0 1.058202 -0.071160 -0.405163

6 0 -1.740484 0.046123 0.023358

8 0 -1.853846 -1.085610 -0.191179

8 0 -1.703366 1.180084 0.250577

6 0 1.713219 1.172325 -0.037173

1 0 1.171912 -0.233932 -1.395539

1 0 1.349632 -1.080718 1.395124

1 0 1.051589 -2.120093 0.004157

1 0 2.646600 -1.364657 0.226563

1 0 2.806688 1.140505 -0.146311

1 0 1.324263 1.987693 -0.645455

1 0 1.487278 1.401351 1.005444

**DMA-CO_2_ TS11 HF = -323.6989268 w(TS) = 1743i**

6 0 1.258768 1.220164 -0.062390

7 0 0.559048 0.000042 0.338336

6 0 1.258721 -1.220203 -0.062100

6 0 -0.909337 0.000004 -0.102165

8 0 -1.282847 -0.000177 -1.234455

8 0 -1.458226 0.000169 1.057562

1 0 0.708001 2.086637 0.298085

1 0 2.255852 1.224204 0.374526

1 0 1.335095 1.271476 -1.150205

1 0 2.255789 -1.224209 0.374853

1 0 0.707891 -2.086569 0.298535

1 0 1.335081 -1.271740 -1.149902

1 0 -0.231366 0.000178 1.360828

**DMA-CO_2_ cis-P1 HF = -323.750357**

6 0 1.426511 -1.150003 0.014997

7 0 0.564735 0.012296 -0.083412

6 0 1.187271 1.315199 0.023618

6 0 -0.789853 -0.175202 -0.012695

8 0 -1.327485 -1.250955 0.016448

8 0 -1.541954 0.951706 0.009932

1 0 0.826302 -2.041870 -0.128423

1 0 2.196791 -1.107851 -0.755931

1 0 1.909373 -1.203838 0.994301

1 0 2.254258 1.203128 -0.151451

1 0 0.820803 2.006302 -0.740175

1 0 1.059546 1.765942 1.012964

1 0 -1.008272 1.746144 -0.013961

**DMA-CO_2_ TS12 HF = -323.7459514 w(TS) = 418i**

6 0 1.462505 -1.101171 -0.056779

7 0 0.559890 0.031245 -0.032962

6 0 -0.778115 -0.194132 0.014552

8 0 -1.540563 0.955303 0.039620

6 0 1.180328 1.335822 0.065106

8 0 -1.287802 -1.283035 0.065198

1 0 0.899555 -2.005763 -0.259879

1 0 2.213957 -0.956279 -0.835057

1 0 1.973183 -1.208989 0.903488

1 0 1.685545 1.597339 -0.868349

1 0 0.438283 2.090999 0.296406

1 0 1.925869 1.326745 0.862975

1 0 -1.617002 1.315966 -0.844677

**DMA-CO_2_ trans-P1 HF = -323.7629283**

6 0 1.438345 -1.139259 0.007876

7 0 0.576216 0.021066 -0.043177

6 0 -0.763359 -0.154862 -0.006944

8 0 -1.446716 1.016851 0.002664

6 0 1.225997 1.313461 0.010941

8 0 -1.335198 -1.223683 0.008538

1 0 0.835504 -2.037052 -0.077660

1 0 2.157397 -1.105460 -0.812727

1 0 1.990087 -1.166917 0.951198

1 0 -2.372946 0.762073 0.014521

1 0 1.977089 1.377085 -0.778619

1 0 0.502578 2.107770 -0.128309

1 0 1.726193 1.453652 0.972986

**(DMA)_2_-CO_2_ TtB2 HF = -458.9354914**

7 0 -2.155493 0.348474 0.630504

6 0 -3.149953 -0.196549 -0.279265

6 0 -1.576593 1.592479 0.147154

7 0 0.530263 -1.120855 0.105631

6 0 1.159080 -2.027697 1.045662

6 0 0.302168 -1.721791 -1.193450

1 0 -2.570851 0.497649 1.538641

1 0 -0.353893 -0.786887 0.480198

1 0 -2.324772 2.367385 -0.068439

1 0 -0.872204 1.978739 0.882926

1 0 -1.023219 1.395602 -0.773034

1 0 -3.953344 0.512335 -0.523275

1 0 -2.663541 -0.482318 -1.213433

1 0 -3.594542 -1.092337 0.152305

1 0 0.604018 -2.966741 1.190435

1 0 2.159966 -2.288542 0.693656

1 0 1.266475 -1.536863 2.012407

1 0 -0.288202 -2.649631 -1.151417

1 0 -0.215687 -1.012264 -1.839210

1 0 1.258088 -1.956098 -1.666722

6 0 1.960440 1.218137 -0.141401

8 0 2.107517 1.315843 1.002939

8 0 1.864667 1.214052 -1.295463

**(DMA)_2_-CO_2_ TS21 HF = -458.9297882 w(TS) = 178i**

6 0 -3.174897 0.566884 -0.579780

7 0 -1.939879 -0.040112 -0.111902

6 0 -2.109151 -0.773731 1.133807

7 0 0.903051 0.741625 0.062349

6 0 1.340277 1.164453 1.384633

6 0 1.436406 1.566338 -1.017322

6 0 1.391184 -1.080947 -0.311705

8 0 0.589234 -1.444702 -1.115134

8 0 2.346286 -1.309662 0.351851

1 0 -1.573886 -0.673172 -0.812639

1 0 -0.121982 0.710615 0.008396

1 0 -2.897273 -1.536074 1.083252

1 0 -1.173932 -1.268834 1.394625

1 0 -2.365229 -0.077876 1.934751

1 0 -3.993215 -0.156563 -0.694191

1 0 -3.499348 1.328089 0.131625

1 0 -3.007073 1.053138 -1.539862

1 0 1.161590 2.617243 -0.895056

1 0 2.523728 1.487627 -1.027020

1 0 1.048373 1.201090 -1.966030

1 0 1.137982 2.225306 1.554175

1 0 0.822265 0.576520 2.139608

1 0 2.408724 0.979226 1.483714

**(DMA)_2_-CO_2_ Dat2 HF = -458.9300139**

6 0 -1.349276 1.600396 -0.910940

7 0 -0.897493 0.663589 0.122557

6 0 -1.374433 1.008142 1.460237

6 0 -1.366596 -0.941079 -0.362631

8 0 -0.584081 -1.305787 -1.207648

8 0 -2.342668 -1.280587 0.245395

7 0 1.899089 -0.043960 -0.095631

1 0 -0.947335 1.279769 -1.868891

1 0 -2.437483 1.587161 -0.954367

1 0 -1.009342 2.613025 -0.690064

1 0 -2.453596 0.879608 1.494308

1 0 -0.926507 0.331238 2.183405

1 0 -1.109824 2.037527 1.706351

1 0 0.136655 0.612979 0.107312

1 0 1.542421 -0.575550 -0.881958

6 0 2.145052 -0.964748 1.005828

6 0 3.082768 0.705329 -0.484055

1 0 3.386001 1.361280 0.333602

1 0 3.936179 0.061325 -0.733104

1 0 2.856600 1.327732 -1.349106

1 0 2.392523 -0.401626 1.907511

1 0 1.244019 -1.546353 1.198446

1 0 2.967422 -1.662774 0.805463

**(DMA)_2_-CO_2_ TS22 HF = -458.9145193 w(TS) = 433i**

6 0 -1.020404 -1.699647 0.707056

7 0 -0.834911 -0.569789 -0.204994

6 0 -1.507837 -0.784763 -1.480744

6 0 -1.183305 0.761636 0.463847

8 0 -0.475921 0.965724 1.471444

8 0 -2.022040 1.435840 -0.107897

7 0 1.634936 0.121854 0.055183

1 0 -0.534589 -1.475125 1.653431

1 0 -2.081885 -1.884346 0.895759

1 0 -0.584793 -2.602436 0.274290

1 0 -2.585845 -0.897598 -1.349240

1 0 -1.340786 0.069670 -2.130365

1 0 -1.106660 -1.685735 -1.949062

1 0 0.589903 -0.321788 -0.237596

1 0 1.298132 0.550805 0.930102

6 0 2.005450 1.175105 -0.896197

1 0 2.176028 0.732644 -1.876230

1 0 1.186249 1.888152 -0.956975

1 0 2.909046 1.689205 -0.569995

6 0 2.659236 -0.896649 0.280832

1 0 2.288959 -1.619482 1.004039

1 0 2.873899 -1.407764 -0.656367

1 0 3.577021 -0.447259 0.659751

**(DMA)_2_-CO_2_ cis-P21 HF = -458.9367339**

6 0 -0.843729 1.661539 -0.404042

7 0 -1.433054 0.480481 0.195847

6 0 -1.272171 -0.780366 -0.355738

8 0 -2.118397 -1.637300 -0.278685

6 0 -2.683397 0.727779 0.886272

8 0 -0.098686 -1.000760 -0.958300

7 0 2.016404 0.157457 0.320724

1 0 -0.037138 1.394396 -1.078210

1 0 -1.592968 2.215841 -0.979572

1 0 -0.452432 2.329932 0.366971

1 0 -3.477321 1.023241 0.192323

1 0 -2.998807 -0.172810 1.402555

1 0 -2.536096 1.529867 1.610777

1 0 1.666769 0.779437 1.037207

1 0 0.634367 -0.477635 -0.538052

6 0 2.613822 -1.015570 0.952322

6 0 2.951443 0.877990 -0.534600

1 0 3.516906 -0.776001 1.525342

1 0 1.885490 -1.482295 1.612918

1 0 2.877408 -1.739483 0.181545

1 0 2.470794 1.768108 -0.938666

1 0 3.865344 1.179487 -0.009681

1 0 3.235090 0.238596 -1.370856

**(DMA)_2_-CO_2_ TS23 HF = -458.9355335 w(TS) = 77i**

6 0 -0.844313 -0.880905 -0.310845

7 0 -1.491717 0.277942 0.024826

8 0 -1.005484 -1.920978 0.286636

8 0 -0.008610 -0.772558 -1.367444

1 0 0.826114 -0.307575 -1.079791

7 0 2.214468 0.315152 -0.272289

1 0 2.962545 0.571736 -0.901496

6 0 2.628610 -0.831940 0.535213

6 0 1.843190 1.469874 0.538647

1 0 2.960159 -1.634413 -0.120562

1 0 3.431418 -0.584253 1.238521

1 0 1.770189 -1.200461 1.097045

1 0 2.640503 1.786339 1.220759

1 0 0.964533 1.216191 1.133064

1 0 1.581078 2.304417 -0.110198

6 0 -1.467011 1.480495 -0.775747

6 0 -2.435626 0.269942 1.120032

1 0 -0.766206 1.369034 -1.594761

1 0 -1.171181 2.337583 -0.164263

1 0 -2.458882 1.685736 -1.189141

1 0 -2.351402 -0.671265 1.652451

1 0 -2.224951 1.097563 1.801858

1 0 -3.459525 0.381201 0.751421

**(DMA)_2_-CO_2_ trans-P2 HF = -458.9516804**

6 0 -3.063757 -1.077045 -0.140271

7 0 -2.167967 0.041115 0.044228

6 0 -0.826339 -0.156665 -0.026893

8 0 -0.115757 0.965405 0.081673

6 0 -2.778083 1.349117 0.128090

8 0 -0.314868 -1.259523 -0.168247

7 0 2.371123 -0.057098 -0.056374

6 0 3.006766 -0.207559 1.244790

6 0 3.305455 0.294888 -1.114320

1 0 -2.491208 -1.998395 -0.133360

1 0 -3.800797 -1.101250 0.665091

1 0 -3.596732 -0.994226 -1.092087

1 0 1.880643 -0.914815 -0.289614

1 0 0.854343 0.707706 0.031852

1 0 -3.528216 1.354389 0.921790

1 0 -2.027504 2.099028 0.347054

1 0 -3.274745 1.607119 -0.812123

1 0 3.821025 -0.942197 1.237651

1 0 3.417461 0.751297 1.564017

1 0 2.262008 -0.520711 1.974788

1 0 3.737514 1.275253 -0.909308

1 0 4.127504 -0.424955 -1.211476

1 0 2.777362 0.350155 -2.065041

**(DMA)_3_-CO_2_ TtB3 HF = -594.1150813**

6 0 -2.473810 -1.544070 1.508475

7 0 -1.623614 -1.225057 0.374666

6 0 -2.131785 -1.757855 -0.878661

7 0 -1.025386 1.734757 -0.336578

6 0 -1.974139 2.734091 0.114493

6 0 -0.866870 1.718589 -1.778996

7 0 1.604331 0.611527 0.768738

6 0 1.287034 0.243147 2.134613

6 0 2.866070 1.319394 0.659370

6 0 1.772652 -1.472057 -0.994344

8 0 1.389937 -2.252297 -0.226102

8 0 2.158891 -0.784709 -1.840525

1 0 -1.321753 0.810488 -0.026356

1 0 -0.692122 -1.583962 0.534919

1 0 0.849171 1.190074 0.404351

1 0 -2.649914 -2.621317 1.633691

1 0 -2.025578 -1.159371 2.424257

1 0 -3.443660 -1.059310 1.382685

1 0 -2.303570 -2.842558 -0.854528

1 0 -3.078985 -1.272427 -1.120964

1 0 -1.430165 -1.536610 -1.682535

1 0 -2.956959 2.653924 -0.374215

1 0 -2.119362 2.642199 1.190697

1 0 -1.585304 3.734792 -0.085963

1 0 -1.812047 1.560312 -2.319674

1 0 -0.446203 2.667676 -2.118354

1 0 -0.169244 0.930100 -2.062229

1 0 2.910988 2.229087 1.276062

1 0 3.685179 0.667462 0.972743

1 0 3.040636 1.596949 -0.379595

1 0 1.323540 1.089638 2.836376

1 0 0.284018 -0.182177 2.173595

1 0 1.988476 -0.514950 2.490576

**(DMA)_3_-CO_2_ TS31 HF = -594.1122395 w(TS) = 185i**

6 0 -2.848257 1.175621 -1.340443

7 0 -1.825646 1.039742 -0.317700

6 0 -2.336895 1.271910 1.024603

7 0 -0.613861 -1.761771 0.151898

6 0 -1.191933 -2.822152 -0.652765

6 0 -0.735122 -2.006326 1.579924

7 0 1.798130 -0.183190 -0.529077

6 0 1.523422 0.173612 -1.910037

6 0 3.163745 -0.626181 -0.295922

6 0 1.327385 1.355476 0.796120

8 0 1.023214 2.246198 0.083908

8 0 1.505864 0.838199 1.838360

1 0 -1.070589 -0.877291 -0.073915

1 0 -1.069324 1.687136 -0.497288

1 0 1.128290 -0.903055 -0.235676

1 0 -3.365368 2.144516 -1.312167

1 0 -2.401313 1.051887 -2.326584

1 0 -3.599388 0.393563 -1.212770

1 0 -2.844360 2.239516 1.134371

1 0 -3.050590 0.487072 1.283582

1 0 -1.518031 1.230025 1.742061

1 0 -2.250254 -3.013814 -0.423680

1 0 -1.111704 -2.568480 -1.709986

1 0 -0.645583 -3.753545 -0.490452

1 0 -1.774038 -2.160836 1.904714

1 0 -0.166106 -2.897791 1.852105

1 0 -0.318059 -1.162871 2.128852

1 0 3.408351 -1.530748 -0.862876

1 0 3.860970 0.159923 -0.589739

1 0 3.297881 -0.826324 0.765428

1 0 1.817789 -0.617323 -2.608138

1 0 0.456474 0.359936 -2.022343

1 0 2.057894 1.088090 -2.168377

**(DMA)_3_-CO_2_ Dat3 HF = -594.1157825**

6 0 -3.298627 -0.594967 1.140498

7 0 -2.146436 -0.653079 0.258610

6 0 -2.505268 -0.956767 -1.120496

7 0 -0.246986 1.681367 -0.023204

6 0 -0.514830 2.824793 0.833799

6 0 -0.237761 2.024330 -1.440731

7 0 1.842718 -0.162324 0.335866

6 0 2.121513 -0.393062 1.756512

6 0 3.034956 0.209336 -0.435848

6 0 0.995213 -1.392593 -0.376863

8 0 0.535737 -2.147255 0.455490

8 0 0.973317 -1.238621 -1.578364

1 0 -0.966117 0.965132 0.131598

1 0 -1.493891 -1.358222 0.581989

1 0 1.139387 0.620988 0.257489

1 0 -3.904844 -1.511305 1.122477

1 0 -2.973260 -0.419275 2.165825

1 0 -3.944444 0.235081 0.846151

1 0 -3.122843 -1.860081 -1.212379

1 0 -3.066104 -0.121071 -1.545333

1 0 -1.596941 -1.099572 -1.704004

1 0 -1.451559 3.337384 0.579348

1 0 -0.575324 2.500033 1.872437

1 0 0.294760 3.552978 0.752768

1 0 -1.175716 2.489158 -1.769406

1 0 0.573106 2.726131 -1.647789

1 0 -0.066864 1.120060 -2.023593

1 0 3.456106 1.134541 -0.043027

1 0 3.777014 -0.584327 -0.365037

1 0 2.746377 0.336616 -1.473867

1 0 2.572025 0.499680 2.190360

1 0 1.191450 -0.627881 2.262622

1 0 2.799007 -1.237207 1.864238

**(DMA)_3_-CO_2_ TS32 HF = -594.1095999 w(TS) = 1013i**

6 0 -2.885155 -0.032072 -0.724651

7 0 -1.799966 -0.280224 0.224475

6 0 -0.822240 -1.378299 -0.212729

8 0 -0.307807 -1.985566 0.726463

6 0 -2.272817 -0.391083 1.602559

8 0 -0.623495 -1.383119 -1.425339

7 0 2.183702 -0.427748 0.193576

7 0 0.050820 1.489333 -0.048296

1 0 -0.923678 0.687925 0.130236

1 0 -2.472974 -0.031095 -1.728317

1 0 -3.647579 -0.811252 -0.656885

1 0 -3.350313 0.931283 -0.508412

6 0 0.078324 2.561542 0.942410

1 0 0.885510 0.870518 0.072559

1 0 1.568214 -1.187991 0.476286

6 0 2.696032 -0.716488 -1.140501

1 0 -2.961330 -1.230472 1.715155

1 0 -1.425195 -0.556213 2.259491

1 0 -2.788673 0.529788 1.880142

6 0 0.014142 1.956861 -1.436137

6 0 3.236892 -0.249908 1.179217

1 0 0.001714 1.084645 -2.086885

1 0 -0.892025 2.539125 -1.604859

1 0 0.881057 2.579088 -1.667681

1 0 -0.846136 3.137459 0.892972

1 0 0.167167 2.126761 1.936236

1 0 0.919193 3.238357 0.776149

1 0 3.200678 0.165401 -1.542747

1 0 3.414088 -1.546151 -1.145510

1 0 1.860205 -0.977803 -1.787495

1 0 3.931436 -1.098836 1.220764

1 0 3.818647 0.643972 0.942803

1 0 2.797442 -0.117872 2.167716

**(DMA)_3_-CO_2_ RI3 HF = -594.1153731**

6 0 -2.979511 0.214488 -0.832428

7 0 -2.029397 -0.225662 0.175813

6 0 -0.961930 -1.105578 -0.277908

8 0 -0.432668 -1.809874 0.604830

6 0 -2.662775 -0.564437 1.438549

8 0 -0.601881 -0.941290 -1.457892

7 0 2.091519 -0.614199 0.102592

7 0 0.327813 1.448483 0.114772

1 0 -0.596167 0.979455 0.251498

1 0 -2.453580 0.404971 -1.762413

1 0 -3.748999 -0.543541 -1.022627

1 0 -3.479342 1.125791 -0.496110

6 0 0.595997 2.373129 1.221714

1 0 1.072677 0.641732 0.122381

1 0 1.347108 -1.307032 0.247933

6 0 2.682073 -0.824383 -1.214456

1 0 -3.389766 -1.377434 1.326067

1 0 -1.907173 -0.883221 2.148839

1 0 -3.189036 0.310384 1.826964

6 0 0.339066 2.070049 -1.220202

6 0 3.059921 -0.704554 1.184888

1 0 0.108721 1.288146 -1.940020

1 0 -0.410218 2.858596 -1.268785

1 0 1.324075 2.491799 -1.412410

1 0 -0.153302 3.162615 1.242437

1 0 0.565901 1.821409 2.158051

1 0 1.582004 2.816306 1.095166

1 0 3.369061 -0.008467 -1.452252

1 0 3.241963 -1.764716 -1.270247

1 0 1.879776 -0.855044 -1.949030

1 0 3.599673 -1.658362 1.184506

1 0 3.796811 0.097204 1.098947

1 0 2.549612 -0.603916 2.142414

**(DMA)_3_-CO_2_ TS33 HF = -594.1148041 w(TS) = 572i**

7 0 -2.077251 -0.151394 0.173396

6 0 -1.031295 -1.032455 -0.279414

8 0 -0.514737 -1.763497 0.597805

8 0 -0.652472 -0.874149 -1.455523

6 0 -2.735408 -0.489537 1.421786

6 0 -2.989190 0.360155 -0.833790

1 0 -1.999333 -0.846727 2.134221

1 0 -3.233717 0.396566 1.821061

1 0 -3.490664 -1.273775 1.288066

1 0 -3.777963 -0.363562 -1.073986

1 0 -3.466704 1.271721 -0.467676

1 0 -2.438201 0.575198 -1.743684

7 0 0.488816 1.423534 0.130103

1 0 -0.461743 1.048053 0.275104

1 0 1.211587 0.458573 0.112219

6 0 0.856671 2.308136 1.236843

6 0 0.541686 2.058476 -1.193370

1 0 0.206790 1.321958 -1.920205

1 0 0.760145 1.766359 2.175103

1 0 1.890714 2.628901 1.116024

1 0 1.567187 2.358272 -1.407027

1 0 0.215005 3.189452 1.264611

1 0 -0.102292 2.937890 -1.226997

7 0 1.977117 -0.715731 0.083132

6 0 2.925084 -0.819619 1.185047

6 0 2.575679 -0.944490 -1.229425

1 0 2.397434 -0.696450 2.129856

1 0 3.431003 -1.789032 1.197793

1 0 3.684677 -0.039898 1.102321

1 0 1.777232 -0.944831 -1.967960

1 0 3.097330 -1.904785 -1.271229

1 0 3.294586 -0.154139 -1.455585

1 0 1.194456 -1.377431 0.227251

**(DMA)_3_-CO_2_ trans-P3 HF = -594.1357168**

6 0 -3.384525 -1.123889 0.738395

7 0 -2.708452 -0.067972 0.022084

6 0 -3.497909 0.661987 -0.943423

6 0 -1.379895 0.142971 0.219556

8 0 -0.717393 -0.507120 1.023218

8 0 -0.874117 1.108444 -0.529944

7 0 1.655728 1.460986 0.085061

6 0 2.414671 2.321114 -0.809631

6 0 1.682397 1.903740 1.473143

7 0 1.875939 -1.511385 -0.083021

6 0 2.713466 -2.582852 0.422189

6 0 1.439726 -1.734096 -1.450690

1 0 1.051107 -1.399098 0.499136

1 0 -2.698093 -1.566128 1.452227

1 0 -4.250925 -0.722867 1.269855

1 0 -3.733845 -1.896083 0.046988

1 0 2.011549 0.502871 0.037762

1 0 0.120782 1.238572 -0.296388

1 0 -4.367053 1.109960 -0.455199

1 0 -2.903023 1.447068 -1.394250

1 0 -3.857565 -0.008628 -1.729282

1 0 2.940924 -2.407136 1.473004

1 0 2.248602 -3.575081 0.330252

1 0 3.657273 -2.606261 -0.126731

1 0 0.930352 -2.697974 -1.591230

1 0 0.754123 -0.939946 -1.747492

1 0 2.299472 -1.711176 -2.124166

1 0 2.700454 1.982882 1.871881

1 0 1.207931 2.883136 1.555404

1 0 1.114082 1.197507 2.076412

1 0 1.947098 3.305681 -0.861496

1 0 3.454422 2.454185 -0.487798

1 0 2.414327 1.892674 -1.811192
